# Editorial: Brain connectivity, dynamics, and complexity

**DOI:** 10.3389/fnhum.2023.1232224

**Published:** 2023-06-19

**Authors:** Stephen José Hanson, Ruben Sanchez-Romero

**Affiliations:** ^1^Rutgers, The State University of New Jersey, New Brunswick, NJ, United States; ^2^Rutgers University, Newark, NJ, United States

**Keywords:** computational neuroscience, brain connectivity, complexity, information theory, dynamics

The contributions in this Research Topic collectively explore the nature of functional brain connectivity and its relation to cognition, emphasizing shared mechanisms and brain complexity. Results presented here reveal common themes in how the brain dynamically reorganizes and adapts to task- and disease-related perturbations.

Some of the contributions present a variety of theoretical and methodological approaches to studying the relationship between connectivity, dynamics, and complexity. The articles include a novel multi-fractal functional connectivity estimation to track changes during visual pattern recognition (Stylianou et al.), a dimensionality reduction technique to facilitate comparisons of oscillatory patterns across task paradigms and modalities (Müller et al.), a new adaptation of the dynamic causal modeling (DCM) approach to capture connectivity changes during more ecological tasks such as movie watching (Nag and Uludag), a new connectivity method based on the Kuramoto model of coupled oscillators applied to network-level fMRI data (Bauer et al.), and a framework to study the relationship between functional connectivity and complexity (Das and Puthankattil).

From an application perspective, contributions focus on the study of the large-scale cortical networks supporting cognition, in health and disease, including navigation subnetworks representing different types of spatial relation representations (Hao et al.), changes in individual differences along the language network hierarchy and their potential as biomarkers (Zhang et al.), the adaptation of the dorsal attention network to demands of a spatial attention task (Machner et al.), the relationship between memory, depression, and inter-network connectivity (Satz et al.), and the recovery of cognitive functions and related task networks after anesthesia (Rokos et al.).

We consider that the integration of these studies has the potential to advance our understanding of common principles governing the dynamic and complex relationship between brain connectivity and cognition.

## Author contributions

All authors listed have made a substantial, direct, and intellectual contribution to the work and approved it for publication.

